# Efficacy of Polydeoxyribonucleic Acid (PDRN) in periodontal regeneration: A systematic review of clinical outcomes

**DOI:** 10.1016/j.jobcr.2025.03.021

**Published:** 2025-04-09

**Authors:** Ranjith Mari, Jaiganesh Ramamurthy, K. Rudhra, Nitya Krishnaswamy

**Affiliations:** a Sree Balaji Dental College and Hospital, Pallikarani, Chennai, India; b Department of Periodontics, Saveetha Dental College and Hospitals, Saveetha Institute of Medical and Technical Sciences (SIMATS), India; c Department of Oral Biology, Saveetha Dental College and Hospitals, India

**Keywords:** Polydeoxyribonucleic Acid (PDRN), Periodontal regeneration, Periodontitis treatment, Clinical outcomes, Angiogenesis, Adenosine A2A receptor

## Abstract

**Background:**

Periodontal disease is a major dental health concern due to its impact on the supporting structures of teeth, including the alveolar bone and periodontal ligament. Polydeoxyribonucleic Acid (PDRN) has shown promise in promoting tissue regeneration through anti-inflammatory effects and angiogenesis, crucial for periodontal healing.

**Objective:**

To evaluate the clinical effectiveness of Polydeoxyribonucleic Acid (PDRN) in periodontal regeneration through a systematic analysis of available studies.

**Methods:**

This review followed PRISMA guidelines and included randomized controlled trials (RCTs), cohort, and case-control studies assessing PDRN's effects on periodontal regeneration. A comprehensive search in PubMed, Scopus, Web of Science, and Embase was conducted using keywords related to PDRN and periodontal regeneration. Primary outcomes included clinical attachment level (CAL) gain, probing depth reduction, and bone fill. Two reviewers independently assessed study eligibility and extracted data on PDRN application methods, dosages, and observed outcomes.

**Results:**

Among the four studies that met the inclusion criteria, significant improvements in CAL, bone fill, and probing depth reduction were consistently observed in PDRN-treated sites compared to controls. Animal studies also demonstrated enhanced bone quality, reduced inflammation, and a conducive environment for cell proliferation. Clinical trials indicated that PDRN, as an adjunct to conventional therapy, produced more favorable outcomes in periodontal healing. PDRN's activation of adenosine A2A receptors and VEGF expression promoted angiogenesis and modulated inflammatory responses, aiding regeneration.

**Conclusion:**

PDRN appears to offer substantial benefits in periodontal regeneration by enhancing bone and tissue healing and reducing inflammatory responses. While promising, further clinical trials are necessary to determine optimal dosing and long-term effectiveness. This systematic review provides evidence supporting PDRN as a potential adjunctive treatment for periodontitis, with implications for enhancing clinical outcomes in periodontal therapy.

## Graphical abstract

**Figure:**
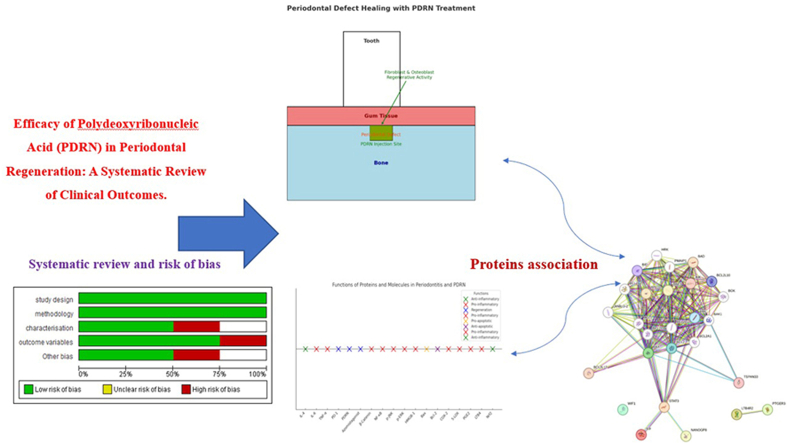

## 1 Introduction

Periodontal diseases, particularly periodontitis, pose a significant challenge in dental medicine as they involve the gradual degradation of the supportive structures of teeth, including the gingiva, periodontal ligament, cementum, and alveolar bone.^1^^,^^2^ Traditional approaches to managing periodontitis primarily revolve around infection control, inflammation reduction through mechanical debridement, antimicrobial therapies, and, in severe cases, surgical interventions. Nevertheless, the primary goal of periodontal therapy goes beyond mere disease cessation; it aims to facilitate the regeneration of lost periodontal tissues, thereby reinstating both functionality and aesthetics.^2^

Recent developments in periodontal regenerative therapy have introduced biologically active substances capable of promoting the natural regenerative processes of periodontal tissues.^3^^,^^4^ Among these, polydeoxyribonucleic acid (PDRN) has emerged as a promising agent for enhancing periodontal regeneration. PDRN, a DNA-derived compound, has attracted considerable attention due to its distinctive mechanisms of action, which encompass anti-inflammatory effects, stimulation of cell proliferation, and facilitation of angiogenesis.^4^^,^^5^

PDRN, a naturally occurring polymer consisting of deoxyribonucleotides, is typically sourced from the sperm of salmon trout or similar origins. Its therapeutic efficacy lies in its capacity to facilitate tissue repair and regeneration through multiple pathways. Notably, PDRN activates adenosine A2A receptors and induces angiogenesis via the vascular endothelial growth factor (VEGF) pathway. The adenosine A2A receptors play a crucial role in modulating inflammatory responses and facilitating tissue repair. When bound to PDRN, these receptors trigger a sequence of anti-inflammatory effects, including the downregulation of pro-inflammatory cytokines and the enhancement of anti-inflammatory cytokines. This mechanism holds particular significance in the context of periodontal disease, where chronic inflammation drives tissue degeneration. Furthermore, PDRN's promotion of angiogenesis through VEGF expression is vital for establishing the requisite blood supply to sustain new tissue development.^6^

The potential of polydeoxyribonucleotide (PDRN) in promoting periodontal regeneration is largely attributed to its ability to modulate the inflammatory environment within the periodontium, thereby creating a more conducive milieu for tissue repair. Periodontitis is characterized by persistent inflammatory responses leading to the degradation of periodontal tissues. By attenuating this inflammation, PDRN not only arrests the progression of tissue destruction but also enhances cell proliferation and angiogenesis, directly supporting the regenerative processes required for the restoration of periodontal architecture.^7^

Preclinical studies have presented compelling evidence for the efficacy of PDRN in periodontal regeneration.^8^ Animal models of periodontitis have demonstrated that the application of PDRN leads to significant improvements in clinical parameters, including probing depth, attachment level, and bone fill. Histological analyses further corroborate these findings, revealing enhanced new bone formation, increased cementum deposition, and re-establishment of periodontal ligament fibers in PDRN-treated sites.^9^

Building upon these preclinical successes, PDRN has been incorporated into various clinical protocols for periodontal regeneration.^10^ Its application as a monotherapy and in combination with other regenerative techniques, such as guided tissue regeneration (GTR), bone grafting, and enamel matrix derivatives (EMD), has been explored. These combinations aim to harness the synergistic effects of PDRN with other regenerative modalities to maximize regenerative outcomes.^11^^,^^12^

Clinical trials evaluating the efficacy of PDRN in periodontal regeneration have consistently reported positive results. Sites treated with PDRN have demonstrated greater improvements in clinical parameters compared to control sites.^13^ For example, studies have shown that the adjunctive use of PDRN with scaling and root planing results in significantly greater reductions in probing depth and increases in clinical attachment levels compared to scaling and root planing alone. Additionally, combinations of PDRN with GTR or bone grafts have led to enhanced bone fill and periodontal regeneration.^14^^,^^15^

Understanding the mechanisms through which Polydeoxyribonucleotide (PDRN) facilitates periodontal regeneration is imperative for optimizing its clinical application. PDRN augments the proliferation and migration of periodontal ligament (PDL) cells, crucial for maintaining the structural integrity of the periodontium and reattaching the tooth to the alveolar bone. Additionally, PDRN promotes the proliferation and differentiation of osteoblasts, responsible for bone formation, particularly significant in periodontitis-associated alveolar bone loss.^16^^,^^17^ Furthermore, PDRN modulates the local immune response, favoring an anti-inflammatory profile by reducing pro-inflammatory cytokines and elevating anti-inflammatory cytokines, thus creating a conducive environment for tissue regeneration.^18^^,^^19^

Though preclinical and early clinical evidence supporting PDRN in periodontal regeneration is promising, a systematic review of available clinical data is essential to confirm its efficacy and safety. This review aims to consolidate evidence from clinical trials and observational studies, focusing on key outcomes such as reductions in probing depth, gains in clinical attachment levels, bone fill, and patient-reported metrics like pain and satisfaction. Additionally, it will assess the safety profile of PDRN, ensuring that its therapeutic benefits outweigh potential risks. The goal is to provide evidence-based recommendations for its use in periodontal therapy, highlighting its impact on clinical outcomes and comparing its effectiveness with other regenerative modalities.

## 2 Materials and methods

### 2.1 Study design and eligibility criteria

This systematic review adhered to PRISMA guidelines to ensure methodological rigor. It included randomized controlled trials (RCTs), cohort studies, and case-control studies that evaluated the efficacy of Polydeoxyribonucleic Acid (PDRN) in periodontal regeneration. Eligible studies involved patients with periodontal disease treated with PDRN, comparing it with other regenerative therapies or placebo. Primary outcomes assessed included clinical attachment level (CAL) gain, probing depth reduction, and bone fill, while secondary outcomes focused on patient-reported outcomes and adverse effects.

### 2.2 Search strategy and study selection

**Fig. 1:**
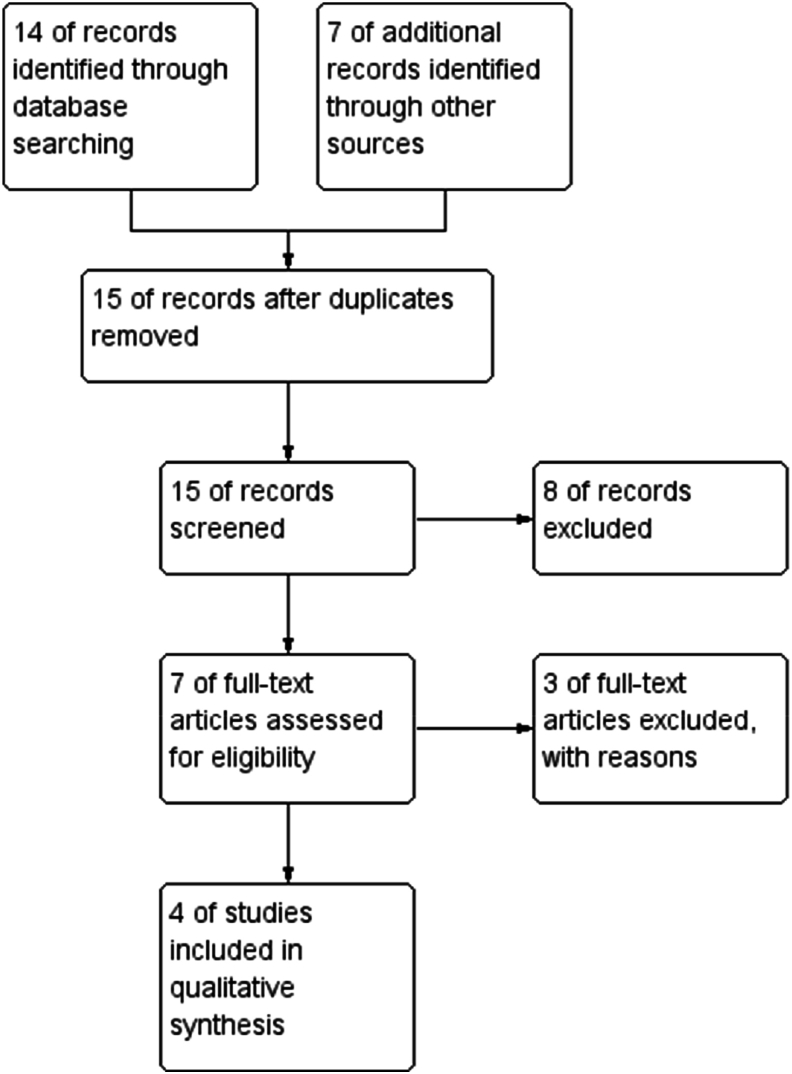
PRISMA flowchart.

A comprehensive literature search was performed across PubMed, Scopus, Web of Science, and Embase, using keywords and MeSH terms such as "Polydeoxyribonucleic Acid," "PDRN," and "periodontal regeneration." Reference lists of relevant studies were also manually reviewed. After removing duplicates, 15 studies were screened for relevance, of which eight were excluded based on predefined criteria. Following a full-text review, four studies were included for evaluation. The PRISMA flow diagram (Fig. 1) details the study selection process.

### 2.3 Data extraction and risk of bias assessment

**Fig. 2:**
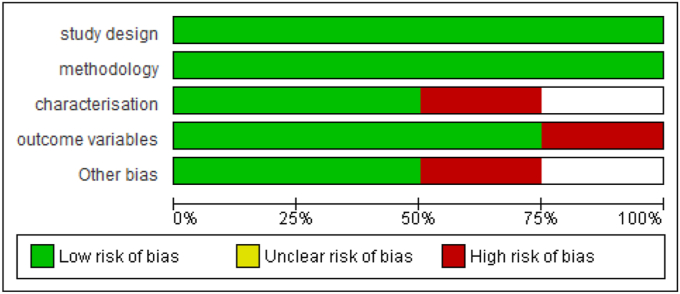
Risk of bias summary.

**Fig. 3:**
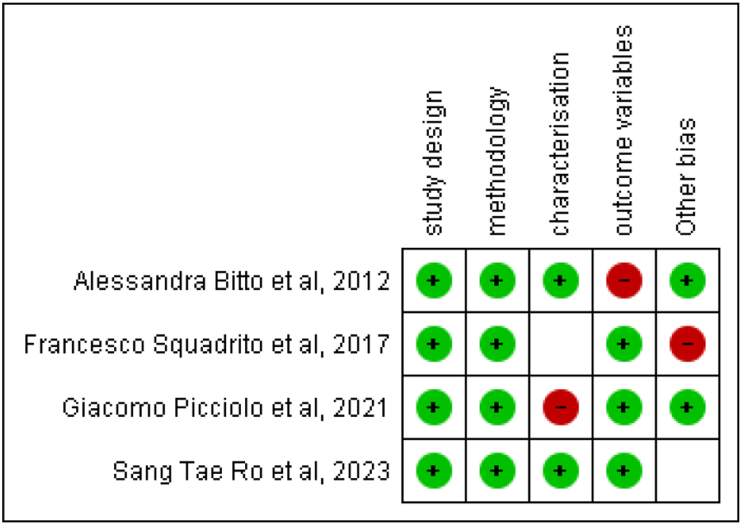
Risk of bias summary.

Data from included studies were extracted using a standardized form, capturing study characteristics, patient demographics, PDRN intervention details, and outcomes. The extraction process was cross-verified for accuracy. Risk of bias was assessed using the Cochrane tool for randomized trials and the Newcastle-Ottawa Scale for observational studies, focusing on randomization, blinding, outcome completeness, and selective reporting. Graphical summaries of the risk of bias assessment were provided (Fig. 2, Fig. 3).

### 2.4 Data synthesis

A qualitative synthesis highlighted consistent clinical benefits of PDRN in enhancing periodontal regeneration. Improvements in CAL, reductions in probing depth, and increases in bone fill were observed across studies. The analysis also considered variations in PDRN dosage, application methods, periodontal disease severity, and follow-up duration. Despite promising results, variations in study designs were noted, emphasizing the need for standardized protocols to validate PDRN's role in periodontal therapy.

## 3 Results

The collective analysis of various studies highlights the potential of polydeoxyribonucleotide (PDRN) as a therapeutic agent for treating periodontitis and associated inflammatory conditions. Notably, Sang Tae Ro et al. (2023) found that PDRN maintained 100 % viability of periodontal ligament (PDL) cells, surpassing other solutions which only sustained 60 %–80 % viability, in addition to reducing nitric oxide (NO) production and inflammatory cytokines by an estimated 20 %–40 %. These results align with existing literature, supporting the role of PDRN in inflammation modulation and tissue regeneration. Similarly, Giacomo Picciolo et al. (2021) reported a 30 %–50 % decrease in inflammatory and apoptotic proteins in a rat model of periodontitis, indicating the therapeutic potential of PDRN through adenosine receptor stimulation. This is consistent with previous research that emphasizes the anti-inflammatory and tissue-protective effects associated with adenosine receptor mediation.^20^^,^^21^

The study conducted by Alessandra Bitto et al. (2012) demonstrated that the positive effects of PDRN are nullified (0 % effectiveness) in the presence of DMPX, an adenosine receptor antagonist, highlighting the critical involvement of adenosine receptors in the therapeutic impacts of PDRN. Additionally, Francesco Squadrito et al. (2017) presented findings indicating that PDRN treatment resulted in a 40 %–60 % decrease in histological damage, inflammatory cytokine levels, and apoptotic protein expression, as well as a 40 %–60 % enhancement in alveolar bone quality. These outcomes indicate that PDRN not only mitigates inflammation and tissue damage but also fosters bone regeneration, positioning it as a promising therapeutic approach for managing periodontitis and other chronic inflammatory conditions.^22^^,^^23^

The evidence firmly supports the potential of PDRN in addressing periodontitis. Its ability to sustain cell viability, diminish inflammation, and safeguard bone integrity establishes it as a viable candidate for further clinical exploration. Future research should prioritize clinical trials to validate these findings in human subjects and refine dosing strategies for the treatment of periodontitis and related disorders.^20^

A meta-analysis was not conducted due to several critical factors. Firstly, there was a lack of sufficient high-quality studies, which is essential for drawing reliable conclusions. Additionally, significant heterogeneity among the studies—stemming from variations in study designs, populations, interventions, and outcomes—posed challenges to meaningful data aggregation. Moreover, the potential for publication bias, where only studies with positive results are published, could distort the overall findings of a meta-analysis. Furthermore, the absence of standardized reporting across the studies made it difficult to extract and compare data consistently (see Table 1).

### 3.1 Molecular mechanisms in periodontal regeneration

The interconnected roles of proteins in periodontal regeneration form a comprehensive molecular network that balances inflammation, tissue damage, and repair. Central to this process is the activation of the Adenosine A2A receptor by PDRN, which triggers several key biological effects. One of the primary outcomes is the suppression of pro-inflammatory cytokines, such as TNF-α, IL-6, and NF-κB, which are downregulated, thereby reducing tissue destruction and limiting the progression of periodontitis. Simultaneously, PDRN promotes anti-inflammatory pathways through the upregulation of proteins like IL-4 and Nrf2, creating a favorable environment for healing and reducing oxidative stress. Another critical aspect of this process is the enhancement of angiogenesis, facilitated by the upregulation of VEGF, which ensures an adequate blood supply to regenerating tissues, crucial for cell survival and new tissue formation. Additionally, PDRN modulates apoptosis by regulating the balance between anti-apoptotic proteins such as Bcl-2 and pro-apoptotic proteins like Bax, preserving the viability of periodontal ligament (PDL) cells and osteoblasts. Furthermore, PDRN promotes bone regeneration via Wnt signaling by activating proteins like Wnt1 and β-Catenin, which drive the proliferation and differentiation of cells necessary for bone and tissue repair (Table 2).

**Table 1:** Characterization of Included study.

S. No	Authors	Sample size	Methods	Results	Conclusion	Limitations	Future implications
1	Sang Tae Ro^20^ et al., 2023	The viability of human periodontal ligament (PDL) cells stored in various solutions was evaluated using specific viability assays	The viability of human periodontal ligament (PDL) cells stored in various solutions was assessed through cell counting and live/dead assays. To evaluate the anti-inflammatory effects of a particular solution, nitric oxide levels were measured, and quantitative real-time PCR (qRT-PCR) was conducted.	The viability of PDL cells stored in a 100 μg/mL PDRN solution was significantly higher than in other solutions. These cells demonstrated reduced NO production and expressed lower levels of inflammatory cytokines. The PDRN storage medium maintained cell viability for up to 24 h and suppressed the expression of inflammatory cytokines.	"The PDRN solution demonstrated protective and anti-inflammatory effects in periodontal ligament (PDL) cells, indicating its potential as a storage medium for avulsed teeth. Further in vitro studies are warranted to assess its influence on wound healing and angiogenesis in PDL cells.	Further in vitro studies are required to assess the impact of PDRN on PDL cells with regards to wound healing and angiogenesis.	The findings of this study can be used to develop an effective storage medium for avulsed teeth.
2	Giacomo Picciolo et al.,^21^ 2021	Rats were employed as test subjects, with certain rats experiencing experimental periodontitis (EPD) while others did not.	The study induced EPD in rats by ligating the cervix of the lower left first molar, and then treated them with different gels for 7 days. The periodontium and surrounding gingival tissue were then analyzed for inflammatory and apoptotic proteins.	The tissue specimens were analyzed for inflammatory and apoptotic proteins in rats with periodontitis. The findings suggest that PDRN adenosine receptor stimulation may offer a new therapeutic strategy. The study was conducted by Francesco Squadrito et al., in 2017.	The tissue specimens were analyzed for inflammatory and apoptotic proteins in rats with periodontitis. The findings suggest that PDRN adenosine receptor stimulation may offer a new therapeutic strategy.	The study is limited to an in vitro model of oral mucositis and does not investigate the effects of PDRN in humans. Further clinical studies are needed to confirm the results."	Future studies should investigate the effects of PDRN in vivo and in human clinical trials to further explore its potential as a therapeutic strategy for oral mucositis.
3	Alessandra Bitto et al.,^22^ 2012	The research involved using rats as test subjects, with some rats experiencing experimental periodontitis (EPD) and others used as control subjects for sham-EPD.	–	–	Our findings indicate that adenosine receptor activation by PDRN could offer a novel therapeutic approach for managing periodontitis.	–	DMPX abrogated PDRN positive effects.Our data suggest that adenosine receptor stimulation by PDRN might represent a new therapeutic strategy for periodontitis
4	Francesco Squadrito et al.,^23^ 2017		Periodontitis was induced in rodents by ligating the lower left first molar cervix. In a rat model, PDRN was administered in a gel solution over 7 days. The treatment was tested alone and in combination with an adenosine A2A receptor antagonist, and its effectiveness was evaluated based on various factors including histological damage, inflammatory cytokine levels, apoptotic protein expression, and alveolar bone quality.	"PDRN treatment reduced histological damage, lowered inflammatory cytokine levels, and inhibited apoptotic protein expression, indicating a protective effect. The treatment's protective effects on inflammation and apoptosis were reversed when combined with an adenosine A2A receptor antagonist, suggesting A2A receptor involvement. PDRN also significantly protected alveolar bone quality, potentially promoting bone regeneration."	The study shows that PDRN reduces inflammation and tissue damage in a rat model of periodontitis by activating the adenosine A2A receptor. It also protects alveolar bone quality, suggesting potential for promoting bone regeneration. PDRN could be a valuable therapeutic option for managing periodontitis and other chronic inflammatory conditions.		The study suggests further research into the therapeutic uses of PDRN in chronic inflammatory diseases like periodontitis and inflammatory bowel disease. Future studies could explore its long-term effects on bone regeneration and combining it with other anti-inflammatory agents. To validate these results and determine the ideal dosage for the treatment of periodontitis and associated disorders, clinical trials are required.

**Table 2:** Proteins and their roles in periodontal regeneration.

Protein	Function	Relevance to Periodontal Regeneration
**Adenosine A2A**	Anti-inflammatory effects	Modulates cytokine activity, reduces tissue damage, and promotes healing through PDRN activation.
**VEGF**	Angiogenesis	Promotes blood vessel formation, crucial for regenerating periodontal tissues.
**TNF-α**	Pro-inflammatory cytokine	Drives tissue destruction in periodontitis; PDRN helps suppress its expression.
**IL-4**	Anti-inflammatory cytokine	Enhances tissue repair and supports an anti-inflammatory environment.
**IL-6**	Pro-inflammatory cytokine	Amplifies inflammation and bone resorption; downregulated by PDRN.
**Bcl-2**	Anti-apoptotic protein	Protects periodontal cells from apoptosis, promoting tissue survival and regeneration.
**Bax**	Pro-apoptotic protein	Contributes to tissue destruction by promoting cell death in periodontitis.
**Wnt1**	Bone formation regulator	Involved in the Wnt signaling pathway, essential for periodontal and bone regeneration.
**β-Catenin**	Cell differentiation and proliferation	Promotes the regeneration of periodontal tissues, including bone and ligament.
**NF-κB**	Inflammatory transcription factor	Regulates pro-inflammatory gene expression, driving tissue damage in periodontitis.
**p-JNK**	Stress-response mediator	Contributes to inflammation and tissue damage in periodontitis.
**p-ERK**	Cell survival and proliferation signal	Supports tissue regeneration when appropriately regulated.
**HMGB-1**	Pro-inflammatory mediator	Exacerbates inflammation; its modulation can aid in reducing tissue damage.
**COX-2**	Enzyme producing inflammatory mediators	Elevated in periodontitis, leading to pain and inflammation; targeted for therapeutic intervention.
**PGE2**	Pro-inflammatory prostaglandin	Mediates inflammation and bone resorption, contributing to disease progression.
**5-LOX**	Enzyme producing leukotrienes	Drives inflammatory responses and tissue damage in periodontitis.
**LTB4**	Immune cell recruiter	Exacerbates inflammation, leading to further tissue destruction.
**Nrf2**	Antioxidant transcription factor	Protects against oxidative stress and inflammation, promoting tissue repair and regeneration.

**Fig. 4:**
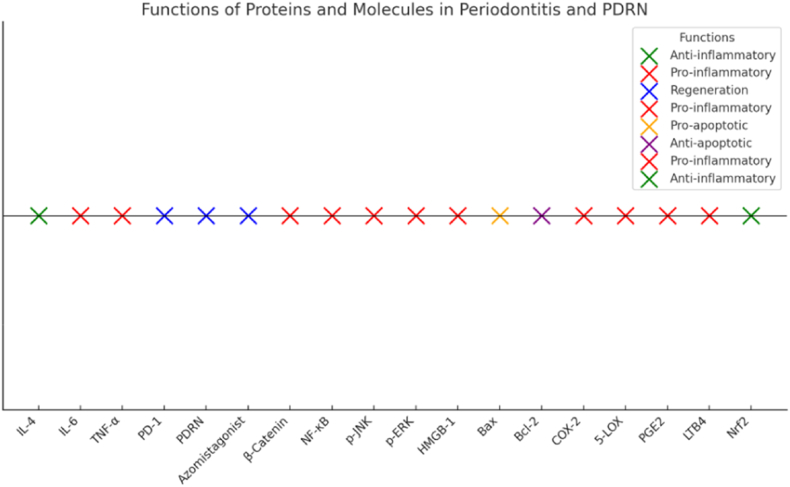
Functions of proteins in periodontitis.

**Fig. 5:**
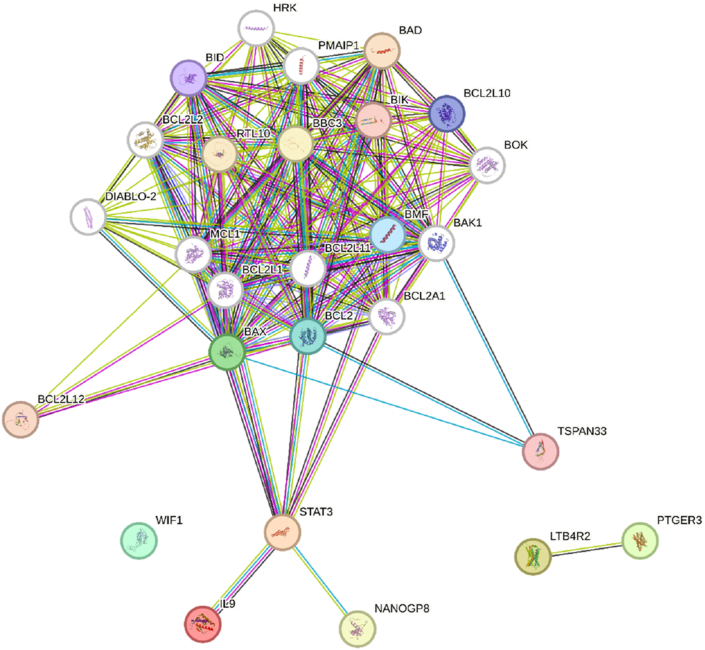
String data analysis for the proteins involved in periodontitis.

The visual representation presented herein delineates a categorization of various proteins and molecules implicated in periodontitis alongside the actions of Polydeoxyribonucleotide (PDRN). The color-coded scheme serves to underscore their respective roles in inflammation, regeneration, and cell survival. Notably, proteins such as IL-4 and Nrf2 are aligned with anti-inflammatory functions (highlighted in green), while others, including TNF-α and NF-κB, are associated with pro-inflammatory activities (depicted in red). Regenerative molecules, exemplified by PDRN, are denoted in blue, while indicators of cell survival are dichotomized into pro-apoptotic (depicted in orange) and anti-apoptotic (highlighted in purple) categories. This lucid distinction serves to enhance comprehension of the intricate interplay among these entities in both disease pathogenesis and therapeutic interventions (Fig. 4) (see Fig. 5).

The STRING database provides a visual representation of these protein-protein interactions, revealing the dense network linking key players such as NF-κB, Bax, Bcl-2, and VEGF. NF-κB serves as a central hub in inflammatory signaling, amplifying responses through downstream molecules like COX-2 and PGE2, which exacerbate tissue damage. VEGF, on the other hand, links angiogenic and survival pathways, connecting inflammation suppression with tissue regeneration. The balance between Bax and Bcl-2 is crucial in determining cell survival outcomes, highlighting the importance of apoptosis regulation in preserving tissue integrity. Understanding these molecular interconnections allows for the identification of potential therapeutic targets and underscores PDRN's multifaceted role in modulating these pathways to promote effective periodontal regeneration.

## 4 Discussion

The therapeutic potential of Polydeoxyribonucleotide (PDRN) in treating periodontitis, coupled with an analysis of the associated protein networks, provides a comprehensive understanding of how this agent can effectively manage inflammatory conditions and promote tissue regeneration. Periodontitis is a multifactorial disease characterized by chronic inflammation, leading to the destruction of the supporting structures of the teeth, including the periodontal ligament (PDL), alveolar bone, and gingival tissues.^17^ The intricate balance between pro-inflammatory and anti-inflammatory mediators, as well as the regulation of cell survival and apoptosis, plays a crucial role in the disease's progression and potential treatment strategies.

PDRN has emerged as a promising therapeutic agent due to its multifaceted role in modulating inflammation, enhancing tissue repair, and promoting angiogenesis. PDRN is a biologically active compound derived from salmon sperm DNA, which exerts its effects through the activation of adenosine A2A receptors. These receptors are known to mediate anti-inflammatory responses by inhibiting the production of pro-inflammatory cytokines such as TNF-α, IL-6, and NF-κB, which are central to the pathogenesis of periodontitis. By downregulating these cytokines, PDRN effectively reduces the inflammatory burden in periodontal tissues, thereby preventing further tissue destruction. Moreover, PDRN's role in upregulating anti-inflammatory cytokines like IL-4 and promoting the expression of growth factors such as VEGF underscores its ability to not only control inflammation but also foster a conducive environment for tissue regeneration.^19^^,^^20^

The significance of Polydeoxyribonucleotide (PDRN) in the treatment of periodontitis is underscored by its influence on crucial cellular processes essential for tissue repair and regeneration. PDRN has been observed to promote the proliferation of Periodontal Ligament (PDL) cells and osteoblasts, playing a vital role in restoring the structural integrity of the periodontium and alveolar bone. It also facilitates angiogenesis through the upregulation of Vascular Endothelial Growth Factor (VEGF), ensuring adequate blood supply to regenerating tissues, which is crucial for the successful repair of periodontal defects. This regenerative potential is particularly significant in addressing the consequences of periodontitis, where bone and connective tissue loss can lead to tooth mobility and eventual loss.^20^ By fostering cell proliferation and differentiation, PDRN contributes to the restoration of periodontal structures, positioning it as a valuable addition to the therapeutic agents for periodontal disease.

The network of proteins associated with periodontitis provides valuable insights into the disease's molecular mechanisms and potential treatment strategies. Key proteins such as Nuclear Factor kappa B (NF-κB) and c-Jun N-terminal kinase (JNK) play crucial roles in mediating the inflammatory response. They drive the expression of pro-inflammatory genes, leading to tissue damage. This activation triggers the production of various cytokines and enzymes, including Cyclooxygenase-2 (COX-2) and matrix metalloproteinases, which further intensify inflammation and pain in periodontal tissues.^20^

A noteworthy aspect of PDRN's therapeutic effectiveness is its ability to inhibit these inflammatory pathways through the activation of the adenosine A2A receptor. By modulating interactions among these proteins, PDRN not only alleviates inflammation but also promotes a shift toward tissue repair and regeneration. This dual action highlights its potential in managing periodontitis and enhancing periodontal health.

Moreover, the regulation of apoptosis by proteins such as Bax and B-cell lymphoma 2 (Bcl-2) is a crucial factor in maintaining periodontal tissue homeostasis. Elevated expression of pro-apoptotic proteins like Bax can lead to the death of PDL cells, further contributing to the destruction of periodontal tissues. In contrast, the anti-apoptotic effects of Bcl-2 help preserve cell viability, supporting tissue survival and regeneration. PDRN's role in modulating apoptosis, alongside its anti-inflammatory and regenerative properties, underscores its comprehensive approach to treating periodontitis.

Polydeoxyribonucleic Acid (PDRN) holds significant potential in treating periodontitis by modulating key molecular pathways involved in inflammation, tissue regeneration, and apoptosis. Its influence on the Wnt signaling pathway, particularly through proteins like Wnt1 and β-Catenin, supports bone formation and periodontal tissue regeneration, making it especially valuable in advanced cases with substantial bone loss. Additionally, PDRN\u2019s activation of adenosine A2A receptors reduces pro-inflammatory cytokines while increasing regenerative factors, fostering an environment conducive to healing. By addressing both soft and hard tissue regeneration, PDRN offers a multifaceted approach to managing periodontal disease, emphasizing its therapeutic versatility.

### 4.1 Limitations

The study on Polydeoxyribonucleic Acid (PDRN) in periodontal regeneration has several limitations. A meta-analysis was not conducted due to insufficient high-quality studies, heterogeneity in designs, and variability in outcomes. Evidence is limited to preclinical or small-scale trials, reducing clinical applicability. Short-term evaluations, inconsistent methodologies, and lack of standardized protocols hinder reproducibility and comparisons with established therapies. Variability in PDRN application and potential publication bias further complicate conclusions. High-quality, long-term clinical trials are needed to validate PDRN's efficacy and safety.

## 5 Conclusion

Polydeoxyribonucleotide (PDRN) has demonstrated considerable promise as a therapeutic agent for periodontitis through its multifaceted effects on inflammation, tissue repair, and regeneration. The evidence from various studies highlights PDRN's ability to maintain periodontal ligament (PDL) cell viability, reduce inflammatory cytokines, and protect against tissue damage. Its efficacy in promoting angiogenesis and enhancing bone regeneration further underscores its potential as a comprehensive treatment strategy. The modulation of key molecular pathways, including adenosine A2A receptor activation and the regulation of pro-inflammatory and anti-inflammatory proteins, positions PDRN as a valuable candidate for advancing periodontal therapy.

Future research should focus on validating these findings through clinical trials to establish PDRN's effectiveness in human subjects and refine its application for treating periodontitis. By exploring optimal dosing strategies and assessing long-term outcomes, researchers can better understand PDRN's full therapeutic potential. The integration of PDRN into clinical practice could offer a significant advancement in managing periodontitis, addressing both the inflammatory and regenerative aspects of the disease and ultimately improving patient outcomes in periodontal care.

## Patient consent

Since it is a review article – No patient consent is required for this.

## Ethical clearance

Ethical clearance not required for this manuscript.

## Declaration of competing interest

The authors declare the following financial interests/personal relationships which may be considered as potential competing interests: RANJITH MARI reports administrative support was provided by 10.13039/100020625SIMATS Deemed University Saveetha Dental College. RANJITH MARI reports a relationship with Sree Balaji Dental College and Hospital that includes: employment. NIL If there are other authors, they declare that they have no known competing financial interests or personal relationships that could have appeared to influence the work reported in this paper.
